# Conceptual Challenges of a Tentative Model of Stress-Induced Depression

**DOI:** 10.1371/journal.pone.0004266

**Published:** 2009-01-30

**Authors:** Bernhard Baune

**Affiliations:** Department of Psychiatry and Psychiatric Neuroscience, James Cook University, Queensland, Australia; Public Library of Science, United States of America

The concept of a stress-induced depression has recently been proposed in light of new findings from animal and human studies. Depression associated with stress involves a number of body systems such as the neuroendocrine and neurotransmitter system and the immune system including cytokines and the dysregulation of the HPA axis interacting in complex pathways. However, numerous research challenges present when addressing a tentative concept of stress-induced depression. One of them is the requirement to establish a causative relationship between stressful environmental factors and stress-related neurochemical and genetic pathways in a complex model of interaction using valid and etiological relevant animal models. Another challenge is the establishment of animal models compatible with the concept of stress-induced depression; however, chronic mild and social stress models are promising models for the study of stressfully perceived environmental events assembling stressors relevant in depression. Moreover, the consideration of individual psychological “neurotic” factors presents another major challenge in animal and human models of stress-induced depression. In addition, the study of translational implications is needed to enhance research into the validity and relevance of a tentative concept of stress-induced depression.

## Stress and Depression

An enduring clinical literature suggests that individual vulnerability to stress and subsequent predisposition to develop certain disease states, notably depression, are related at least in part to a history of early environmental adversity. Exposure to early trauma, for example sexual and physical abuse or other types of early disadvantage, can increase several-fold the risk of being diagnosed with a depressive illness in adulthood [1], [2].

Similarly, the onset and recurrence of adult depression can reliably be predicted by the presence of environmental stressors, often labeled “life events.” Some individuals may have a genetic propensity to select themselves into high-risk environments, but epidemiological studies using identical and non-identical twins have shown that there is still a substantial causal relationship between stressful life events and depression [3].

Since the mechanisms by which stress is mediated in the central nervous system are multiple and include the autonomic nervous system, the neuroendocrine and neurotransmitter systems, and the immune system, it appears challenging to identify a single “stress”-pathway leading to or causing depression. However, it is obvious that stress may have an impact on a number of other systems relevant to depression, including the autonomic nervous system, the neuroendocrine system, and the immune system. In addition, stress is related to symptom clusters such as sleep disturbance, impaired learning, and impaired memory, which have been suggested to form endophenotypes of depression [4].

## Specific Stress Models Relevant to Depression

The study of the relationship between stress and depression depends on the concepts and models used for defining stress and depression. Clinical studies have consistently implicated abnormalities in the regulation of key neuroendocrine responses to stress in a proportion of patients with depression, with a hyperactivity of the HPA axis that is probably driven by hypersecretion of the hypothalamic peptide corticotropine releasing hormone (CRH) [5], [6]. Certain areas of the brain, including parts of the hippocampal formation, are more sensitive to damage from high levels of glucocorticoids [7].

Inflammation and cytokines appear to play an important role in mediating the relationship between stress and the development of depression and indicate the complex relationship between stress and the immune and neuroendocrine systems. In humans, psychological stress significantly increases pro-inflammatory (but inhibits anti-inflammatory) cytokine production in patients responding to stress and anxiety. In depressed patients, increases in macrophage activity and the production of pro-inflammatory cytokines complement, and some acute-phase proteins have been consistently reported [8].

Furthermore, animal experiments have demonstrated that pro-inflammatory cytokines, such as interleukin (IL)-1beta, IL-6, and TNF-alpha can stimulate the hypothalamus to release corticotrophin releasing hormone (CRH), which, via adrenocorticotropic hormone (ACTH), induces glucocorticoid (GC) secretion. Excessive secretion of GC may downregulate GC receptors in the hippocampus, which impairs the GC feedback system. Similar neuroendocrine changes also occur in depressed patients. From the neurotransmitter perspective, pro-inflammatory cytokines have been found to reduce both serotonin and norepinephrine availability to the brain to levels similar to those observed in depression [9].

There are a number of animal stress models of depression, including learned helplessness, which is perhaps the best-known stress model of depression; other models are the inescapable foot shock and intracranial-self stimulation model, the behavioural despair model, and the chronic unpredictable mild stress and the social stress models of depression. All models have presented with significant validity problems relevant to a hypothesized etiological stress model of depression. A promising group of stress models of depression is called “chronic social stress models” [10], which is considered a model of social defeat or subordination [11], and therefore may mimic situations occurring in humans and may be an appropriate model for depressive disorders [12]. It is suggested that the rat chronic social stress model may be useful to describe depressive disorders; however, further research into the context of the immune system [13] and the neuroendocrine and neurotransmitter systems is required to explore the validity of the chronic social stress model in the context of stress-induced depression.

## Conceptual Challenges

Although a tentative new subtype of depression has been proposed and called *stress-induced depression* [14], the scientific question of whether stress can cause depression consistent with existing diagnostic criteria such as in *DSMIV* (*Diagnostic and Statistical Manual of Mental Disorders,* 4^th^ edition) or *ICD10* (*International Statistical Classification of Disease and Related Health Problems,* 10^th^ revision) is unresolved. Is it possible that stress can be a causative factor in depression, or should the research approach be more precise to unravel the stress-induced specific molecular mechanisms eventually inducing symptoms that are defined as depression? In the latter case, the term *stress-associated depression* might be better-suited to describe the complex interactions between environmental stress and molecular mechanisms in a complex phenotype of depression. In line with this is the observation that individuals developing “stress-associated depression” are characterized by a genetically and socially determined higher susceptibility to stress. Diathesis-stress theories of depression predict that genes influence individuals' sensitivity to stressful events, consistent with a potentially important role of gene-by-environment interactions played in the etiology of depressive psychopathology [15].

Although the concept of a clinically relevant stress-induced depression, which is characterized by a heterogeneous phenotype, intuitively may have clinical application and a relatively low threshold of acceptance from a clinical point of view as well as from a basic science perspective into the molecular mechanisms of stress, the definition of a circumscribed and specific phenotype of stress-induced depression is lacking. Given the lack of specificity between stressors and psychopathological outcomes [16], one may hypothesize that gene–stressor interactions account for a better outcome specificity than stress alone. Therefore, psychopathological constructs reflecting gene-by-environment interactions might be among the most specific and most useful endophenotypes for major depression. As an example, Caspi et al. [17] have shown in a representative prospective study that 5-HTT genotypes moderate the influence of stressful life events on major depression.

The establishment of a causative relationship between stress and a phenotype of depression is most challenging as it requires a chain of evidence linking a number of crucial factors built into complex systems: (1) the environmental factor of stress, (2) the individual perception of and vulnerability to stress for which the diagnostic construct of neuroticism defined as general vulnerability to anxiety and depressive symptoms under stress might be useful, (3) the genetic level and corresponding (4) neurochemical/neuroanatomical characteristics of stress-induced changes, and (5) the psychopathology phenotype consistent with symptoms of depression.

In light of such complexity, the establishment of a causative relationship in a concept of stress-induced depression is still facing conceptually and methodologically unresolved problems, some of which have been discussed in this Overview.

## Conclusions

Since stressfully perceived environmental events activate a number of neurochemical systems including the immune system in a complex interaction of pathways in the individual, it appears difficult at this stage to define a homogenous psychopathological and neurochemical endophenotype required for a model of stress-induced depression. Research in this area is required to establish a causative relationship between stressful environmental factors, individual psychological “neurotic” factors, and stress-related neurochemical and genetic pathways. The development of adequate human and animal models and the study of their translational implications will enhance the research into the validity and relevance of a concept of stress-induced depression.

In the *PLoS ONE Special Collection* “Stress-Induced Depression and Comorbidities: From Bench to Bedside,” some of the conceptual challenges relevant for the scientific discussion of a concept of stress-induced depression will be addressed by presenting empirical data from animal and human studies.
